# Regulation of Rho GTPases in the Vasculature by Cullin3-Based E3 Ligase Complexes

**DOI:** 10.3389/fcell.2021.680901

**Published:** 2021-05-31

**Authors:** Fabienne Podieh, Peter L. Hordijk

**Affiliations:** Department of Physiology, Amsterdam UMC, Amsterdam, Netherlands

**Keywords:** Cullin3, ubiquitin, endothelial cells, vasculature, signaling, Rho GTPases

## Abstract

Cullin3-based ubiquitin E3 ligases induce ubiquitination of substrates leading to their proteasomal or lysosomal degradation. BTB proteins serve as adaptors by binding to Cullin3 and recruiting substrate proteins, which enables specific recognition of a broad spectrum of targets. Hence, Cullin3 and its adaptors are involved in myriad cellular processes and organ functions. Cullin3-based ubiquitin E3 ligase complexes target small GTPases of the Rho subfamily, which are key regulators of cytoskeletal dynamics and cell adhesion. In this mini review, we discuss recent insights in Cullin3-mediated regulation of Rho GTPases and their impact on cellular function and disease. Intriguingly, upstream regulators of Rho GTPases are targeted by Cullin3 complexes as well. Thus, Rho GTPase signaling is regulated by Cullin3 on multiple levels. In addition, we address current knowledge of Cullin3 in regulating vascular function, focusing on its prominent role in endothelial barrier function, angiogenesis and the regulation of blood pressure.

## Introduction

Ubiquitination is an important post-translational modification, which controls a variety of cellular functions. It entails the covalent attachment of the highly conserved, 76 amino acid ubiquitin peptide to lysine residues of a substrate resulting in different cellular responses. Ubiquitin itself can also be ubiquitinated, leading to various types of di- or polyubiquitin chains. Whereas, as an example, K48-linked ubiquitin chains target the substrate for proteasomal degradation, K33 or K27 linkages have been associated with protein trafficking and DNA damage response, respectively. Regulation of protein stability by ubiquitination controls cell cycle progression, endocytosis, signal transduction or transcriptional regulation. In addition, ubiquitination affects protein activity, localization and protein-protein interactions. Mechanistically, E1, E2, and E3 ubiquitin ligases act in a cascade, with the E3 ligase being essential for specific interaction with the substrate protein (Hershko et al., 1983; Hershko and Ciechanover, 1998; Komander and Rape, 2012; Popovic et al., 2014; Akutsu et al., 2016).

**FIGURE 1:**
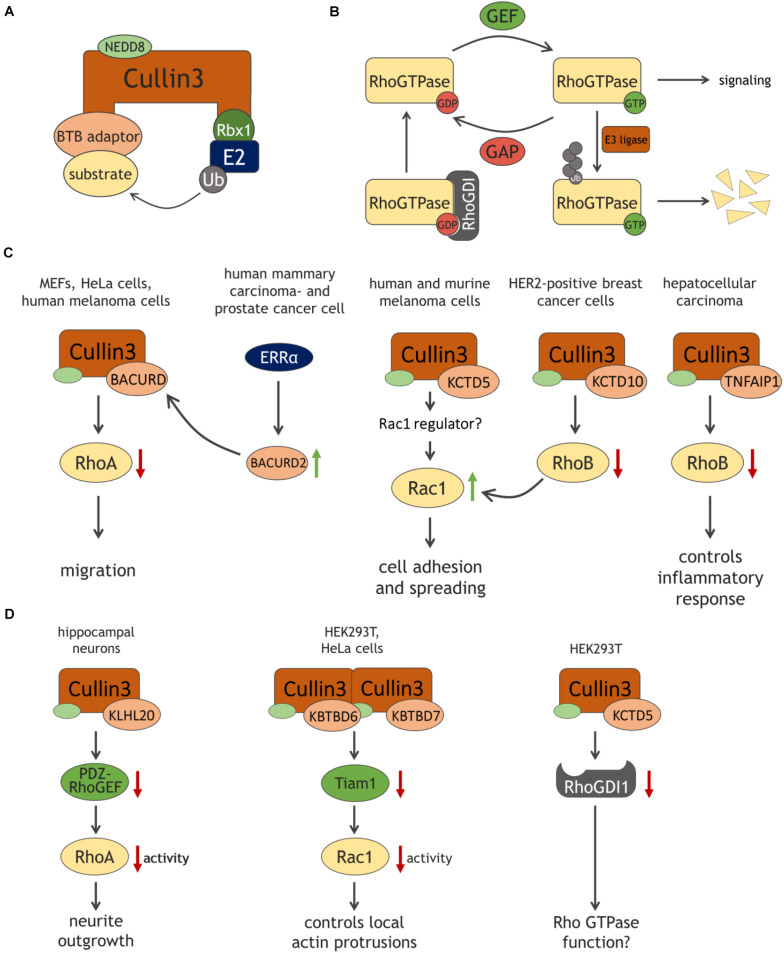
Schematic representation of the Cullin3 E3 ligase complex **(A)** and of the Rho GTPase cycle **(B)**, with ubiquitination as an additional level of control of GTPase expression and signaling output. **(C,D)** Cullin3-BTB complexes target Rho GTPases and their regulators in various cell types. See text and list of abbreviations for details.

Among the different families of E3 ligases, RING ubiquitin ligases comprise the largest subgroup (Metzger et al., 2014). Within a Cullin-based RING ligase complex, Cullin proteins act as scaffolds, interacting through their C-terminus with the small protein Rbx1 or Rbx2, which provides the catalytic domain and recruits the ubiquitin-bound E2 ligase (Ohta et al., 1999; Figure 1A). Conversely, adaptor proteins interact with the N-terminus of Cullins and recruit substrate proteins to the E3 ligase complex (Petroski and Deshaies, 2005; Lydeard et al., 2013). Finally, Cullin3 ligase activity requires the covalent attachment of the ubiquitin-like molecule NEDD8 (neddylation), a modification which facilitates substrate binding and –ubiquitination (Sakata et al., 2007; Duda et al., 2008).

BTB domain-containing proteins are typical substrate-binding subunits of Cullin3 E3 ligases (Furukawa et al., 2003; Xu et al., 2003; Pintard et al., 2004). Approximately 200 genes encoding BTB proteins have been identified (Stogios et al., 2005), of which only 38 have been reported to assemble into a Cullin3-based E3 ligase complex (Wang et al., 2020). The large number of BTB proteins allows Cullin3 ubiquitin ligases to recruit a wide range of proteins as substrates, and participate in diverse cellular pathways, including mitosis, cytokinesis, autophagy, and apoptosis, protein-trafficking and stress responses (Genschik et al., 2013; Cheng et al., 2018; Wang et al., 2020).

The large family of small GTPases is divided into several subfamilies according to sequence, structure and function (Wennerberg et al., 2005). Rho GTPases form a smaller subgroup of 22 members with diverse functions in healthy as well as diseased cells and tissues. Rac1, RhoA/B and Cdc42 represent the classical, best studied members of the Rho GTPase subfamily. These Rho GTPases act as key regulators of actin cytoskeleton dynamics and control fundamental cellular functions such as adhesion, migration and polarity. Whereas Rac1 activity facilitates the formation of a peripheral F-actin meshwork pivotal for cell spreading and adhesion, RhoA/B proteins promote assembly of contractile F-actin stress fibers leading to cell contraction (Hall, 1998; Jaffe and Hall, 2005). Aberrant Rho GTPase signaling, mainly due to increased GTPase activity, has been associated with diseases such as cancer and neurodegenerative disorders (Cook et al., 2014; Haga and Ridley, 2016; Arrazola Sastre et al., 2020).

Rho GTPases act as binary molecular switches being either in an inactive, GDP-bound state or an active, GTP-bound state (Figure 1B). This cycling is tightly regulated by guanine nucleotide exchange factors (GEFs) catalyzing the exchange of GDP to GTP, and GTPase-activating proteins (GAPs), which accelerate GTP hydrolysis thereby inactivating the GTPase. Guanine dissociation inhibitors (GDIs) inhibit Rho GTPase activation by binding the inactive form and preventing GDP dissociation (Bos et al., 2007; Cherfils and Zeghouf, 2013). GTPase activity is additionally controlled by several post-translational modifications such as ubiquitination. The E3 ligase Smurf1 targets RhoA for degradation thereby regulating cell polarity, whereas Rac1 stability is modulated by the E3 ligases HACE1 or cIAP/XIAP controlling cellular spreading and migration (Wang et al., 2003; Torrino et al., 2011; Oberoi et al., 2012; Daugaard et al., 2013).

In this mini review, we summarize current knowledge on Cullin3 complexes targeting Rho GTPases, controlling GTPase activity and contributing to Rho GTPase-related diseases. In addition, we focus on the regulation of vascular function in health and disease by Cullin3 ligase complexes.

## Regulation of Rho Gtpases by Cullin3

### Cullin3-Rho GTPases in Cell Adhesion, Migration and Cancer

Next to Rho GTPase activity being regulated by GEFs, GAPs and RhoGDI, ubiquitination of (active) Rho GTPases has emerged as an important layer of additional control. An example is the ubiquitination of active, but not inactive, Rac1 by HACE1 or XIAP/cIAP (Torrino et al., 2011; Oberoi et al., 2012). Consequently, Cullin3-based E3 ligases play an important, albeit poorly understood, role in regulating Rho GTPase signaling toward F-actin polymerization and stress fiber formation driving cell adhesion and migration in a variety of cell types.

Chen et al. (2009) were the first to show that RhoA is targeted by a Cullin3-based ubiquitin E3 ligase and they discovered that a well-conserved family of BTB-domain proteins named BACURDs serve as substrate adaptor proteins (Figure 1C). *In vitro* ubiquitination assays suggested the preference for RhoA-GDP as substrate of Cullin3-BACURD, limiting the pool of RhoA-GDP potentially being activated by RhoGEFs. Loss of either Cullin3, BACURD1 or BACURD2 results in impaired RhoA degradation and an increase in RhoA activity, abnormal stress fiber formation and migration defects in MEFs and HeLa cells (Chen et al., 2009). In Xenopus laevis embryos, BACURDs are essential for RhoA-mediated convergent cell movements during gastrulation (Chen et al., 2009). These findings indicate that the Cullin3 complex controls cell migration by maintaining appropriate levels of RhoA.

The Bardet-Biedl syndrome (BBS) is a genetic disorder with multiple systemic manifestations. The missense mutation M390R in the BBS1 protein is carried by 80% of Caucasian patients (Mykytyn et al., 2003; Cox et al., 2012). BBS proteins are involved in directional cell migration by participating in the formation and function of cilia, microtubular projections from the cell surface. Fibroblasts from patients or from mice carrying the BBS1 M390R mutation display abnormal orientation of cilia and migration defects, accompanied by reduced Cullin3 levels and elevated RhoA expression and activity. Notably, pharmacological inhibition of the Rho effector Rho kinase (ROCK) rescued the migration defect, while inhibition of Cullin3 by MLN4924 (a compound that prevents neddylation and activation of Cullin complexes) blocked cell migration (Guo and Rahmouni, 2019). These data support the finding of Cullin3-mediated control of RhoA turnover and cell migration (Chen et al., 2009). In addition these data suggest that RhoA-mediated migration of fibroblasts is dysregulated by Cullin3 in patients with a BBS1 M390R mutation, potentially contributing to disease.

Regulation of RhoA stability by Cullin3 ubiquitin ligases is also observed in tumor cells. The transcription factor estrogen-related receptor α (ERRα) stimulates the expression of BACURD2, which, in complex with Cullin3, regulates RhoA stability and activity (Figure 1C). This complex thus controls cell migration in human mammary carcinoma- and prostate cancer cells. Since high expression of ERRα correlates with poor prognosis of various tumors, this suggests that ERRα-controlled BACURD2 expression and consequent regulation of RhoA-dependent migration contributes to the invasive phenotype of cancer cells (Sailland et al., 2014). In the human melanoma cell line A375, silencing of Cullin3 results in elevation of RhoA protein levels, indicating that a Cullin3 E3 ligase complex regulates RhoA in this cell type as well (Vanneste et al., 2020). In contrast, the Cullin3-KCTD10 complex does not target RhoA for degradation in HER2-positive breast cancer cells (Murakami et al., 2019). Thus, these findings suggest that, depending on the tumor cell type, Cullin3 and its associated substrate adaptor serve as modulators of RhoA signaling, potentially contributing to invasive behavior.

Cullin3, via the adaptor protein KCTD5, also regulates cell adhesion and spreading in murine melanoma cells, suggestive of Rac1 activation. However, Rac1 levels are not affected by loss of KCTD5, indicating that Cullin3-KCTD5 regulates Rac1 activity in an indirect manner (Canales et al., 2020; Figure 1C). Consistent with this, Vanneste et al. showed in a human melanoma cell line that the loss of Cullin3 itself increases Rac1 activity (Vanneste et al., 2020). Similarly, Rac1 protein levels remained unaffected, supporting the idea that Cullin3-based E3 ligases control Rac1 regulators, indirectly affecting Rac1 signaling (Vanneste et al., 2020). An example of such indirect regulation is the targeting of the Rac1-specific GEF Tiam1 by Cullin3-KBTB6/KBTBD7 (Genau et al., 2015) (see below). However, in HER2-positive breast cancer cells Rac1 activity could also be increased due to Cullin3-KCTD10-mediated loss of RhoB. Cullin3 may directly regulate RhoB turnover allowing membrane translocation and activation of Rac1 (Murakami et al., 2019). This notion is in line with a previous study showing that RhoB suppresses Rac1 endosomal trafficking to the membrane, thus preventing Rac1 activity (Marcos-Ramiro et al., 2016).

RhoB stability is not only regulated by Cullin3 in HER2-positive breast cancer but also in hepatocellular carcinoma. In complex with Cullin3, the adaptor TNFAIP1, also known as BACURD2, targets RhoB for degradation. Depletion of TNFAIP1 results in RhoB accumulation and expression of pro-inflammatory IL-6 and IL-8 genes, suggesting that the Cullin3-TNFAIP1-RhoB axis controls inflammatory response in hepatocellular carcinoma cells (Liu et al., 2021; Figure 1C).

### Targeting of Rho GTPase Regulators by Cullin3

Besides targeting Rho GTPases directly, Cullin3 complexes alter Rho GTPase signaling indirectly via targeting regulatory proteins (Figure 1D). Cullin3-KLHL20 promotes ubiquitination of brain-enriched PDZ-RhoGEF in hippocampal neurons, preventing activation of RhoA and promoting neurite outgrowth (Lin et al., 2011). Similarly, protein levels of the Rac1-specific GEF Tiam1 are regulated by a Cullin3 complex. Here, Cullin3 forms a heterodimeric complex with the adaptor proteins KBTBD6 and KBTBD7. Deficiency of KBTBD6 or KBTBD7 leads to an elevation of Tiam1 protein levels and Rac1 activity, resulting in an increase of cortical actin and actin protrusions (Genau et al., 2015). Intriguingly, binding to GABARAP proteins is crucial for the Cullin3-KBTBD6/KBTBD7 complex. Since GABARAP proteins are covalently attached to lipid membranes, they may recruit the Cullin3-KBTBD6/KBTBD7 complex to intracellular or plasma membranes, locally controlling Tiam1 ubiquitination and restricting Rac1 activation (Palamidessi et al., 2008; Genau et al., 2015).

Recently, it was shown that Cullin3 and the adaptor protein KCTD5 form a complex with RhoGDI1, promoting its ubiquitination (Cho et al., 2020). While these authors reported that depletion of KCTD5 attenuates RhoGDI degradation in HEK293T cells, RhoGDI levels are unaffected when Cullin3 is silenced in human melanoma cells (Vanneste et al., 2020). Whether RhoGDI is a direct target of Cullin3 E3 ligase in a cell-specific manner and if modulation of RhoGDI stability by Cullin3-KCTD5 has functional consequences for Rho GTPases remains to be established.

By directly regulating Rho GTPases, but also by targeting their regulators such as GEFs and RhoGDI, Cullin3-based E3 ligases are important gatekeepers of Rho GTPase function. It is unknown whether stability of RhoGAP proteins are also affected by Cullin3-based E3 ligases and BTB adaptor proteins.

## Cullin3 and Vascular Function

The inner layer of blood vessels is formed by endothelial cells (ECs), which are tightly connected and function as dynamic barrier, regulating the extravasation of leukocytes and plasma from the circulation to the surrounding tissue. To preserve endothelial integrity, dynamic control of endothelial cell-cell contacts is crucial. The adhesion of neighboring ECs is mainly mediated by the junctional protein VE-cadherin and -associated cytoskeletal dynamics. As key regulators of the actin cytoskeleton, Rho GTPases play an essential role in endothelial integrity (Marinkovic et al., 2015; Komarova et al., 2017; Pronk et al., 2019).

**FIGURE 2:**
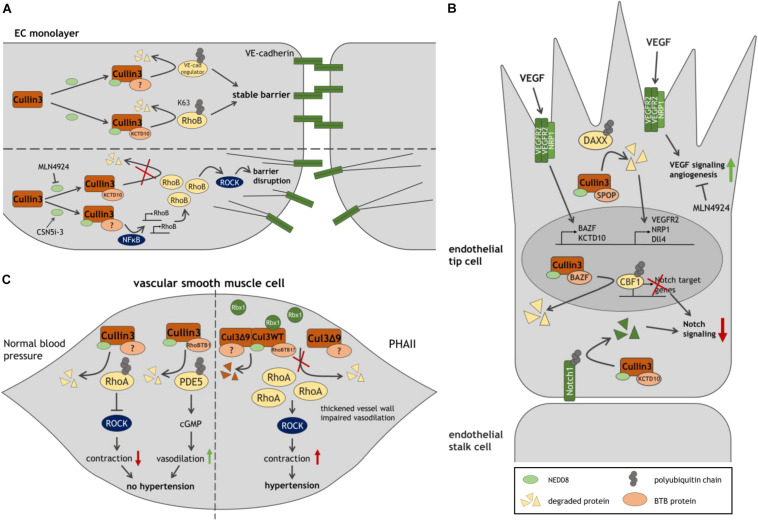
Model of Cullin3 ligases in vascular function. **(A)** Role of Cullin3 in stable and disrupted endothelial barriers, through its regulation of RhoB-ROCK-mediated contractility. **(B)** The different functions of Cullin3 ligases in endothelial tip cells, driving angiogenesis. **(C)** By regulating RhoA- and PDE5-mediated signaling in SMC, Cullin3 controls SMC function and blood pressure. The Cullin3 mutant Cul3Δ9 interferes with the regulation of RhoA, resulting in SMC contraction and hypertension. See text and list of abbreviations for details.

The first evidence that Cullin3 is required for endothelial barrier function is reported by Sakaue et al. (2017a). Inhibition of Cullin activity by blocking its neddylation using MLN4924 or by silencing Cullin3 in human umbilical vein ECs (HUVECs) leads to disruption of endothelial cell-cell contacts and increases vascular permeability. Concomitantly, VE-cadherin half-life is markedly declined by Cullin3 depletion. These findings suggest that Cullin3 targets a negative regulator of VE-cadherin for proteasomal degradation, stabilizing VE-cadherin and endothelial cell-cell contacts (Sakaue et al., 2017a; Figure 2A). An additional study showed that this effect of Cullin3 inhibition is attributed to elevated levels and activity of RhoB, inducing F-actin stress fiber formation and EC contractility. The Cullin3-KCTD10 E3 ligase complex specifically K63-polyubiquitinates RhoB on two lysine residues (Lys 162/Lys 181) driving lysosomal degradation of RhoB. Thus, continuous Cullin3-mediated ubiquitination limits RhoB activity, representing a key mechanism to stabilize endothelial barrier integrity (Kovacevic et al., 2018).

Whereas MLN4924 blocks neddylation and activation of Cullin proteins, the inhibitor of the COP9 signalosome (CSN)5i-3 prevents removal of NEDD8 and stabilizes Cullin activity, potentially protecting endothelial integrity, e.g., during inflammation (Schlierf et al., 2016). Unexpectedly however, CSN5i-3 was found to trigger an NFκB-mediated inflammatory response in HUVECs resulting in the disruption of junctional integrity. An induction of vascular leakage by CSN5i-3 was also confirmed in zebrafish embryos. Mechanistically, NFκB strongly promotes the *de novo* synthesis of RhoB and partly of RhoA, followed by ROCK-mediated cell contraction (Majolee et al., 2019).

Cullin3 is not only involved in endothelial barrier function, but also plays an essential role in angiogenesis, the formation of new blood vessels from preexisting vasculature. Angiogenic sprouting is led by migratory tip cells followed by proliferative stalk cells. Vascular endothelial growth factor (VEGF) and VEGF receptor 2 (VEGFR2) signaling determine specification of ECs into tip cells, while active Notch provides anti-angiogenic signals and promotes stalk cell fate. Thus, a strict balance between VEGF-VEGFR2 and Notch signaling is essential for angiogenesis (Eilken and Adams, 2010; Blanco and Gerhardt, 2013).

Blocking activation of Cullins by MLN4924 suppresses angiogenesis *in vitro* and *in vivo* (Yao et al., 2014; Shi et al., 2020). Intriguingly, it has been reported that VEGF promotes expression of the two BTB proteins BAZF and KCTD10 in HUVECs (Ohnuki et al., 2012; Ren et al., 2014; Figure 2B). In a complex with Cullin3, BAZF promotes polyubiquitination and degradation of CBF1, a Notch-related transcription factor (Ohnuki et al., 2012). Conversely, Culin3-KCTD10 targets the Notch receptor Notch1 for degradation (Ren et al., 2014). These data suggest that Cullin3 functions in two distinct complexes restricting Notch signaling and controlling VEGF/Notch cross-talk during angiogenesis. In line with that notion, BAZF^–/–^ mice show upregulation of Notch signaling and reduced vessel sprouts and branches in the developing retina (Ohnuki et al., 2012). KCTD10-deficient mouse embryos or -HUVECs exhibit upregulation of Notch1. Since KCTD10^–/–^ mice display severe defects in angiogenesis and heart development leading to embryonic lethality, KCTD10 may play an essential role in embryonic angiogenesis and cardiovascular development (Ren et al., 2014).

In line with these findings, the Cullin3-SPOP complex has been proposed as regulator of VEGFR2-mediated endothelial cell function. By targeting the substrate DAXX for degradation, the E3 ligase facilitates expression of VEGFR2 in HUVECs. Depletion of Cullin3 or SPOP abrogates VEGFR2 expression as well as the VEGF-A induced angiogenic response, whereas DAXX silencing rescued VEGFR2 levels during SPOP depletion. Similarly, the Cullin3-SPOP-DAXX axis acts as a regulator of angiogenesis by positively controlling expression of the VEGFR2 co-receptor NRP1 and Notch ligand Dll4, which is typically expressed in tip cells (Blanco and Gerhardt, 2013; Sakaue et al., 2017b). The reported studies suggest multiple (pro-angiogenic) roles for Cullin3 in angiogenesis in an adaptor protein-dependent manner and might provide new strategies to treat angiogenesis-related diseases.

Emerging evidence exists that Cullin3 also functions as a regulator of blood pressure (Figure 2C). RhoA and the Rho effector ROCK regulate vascular tone and hypertension (Loirand and Pacaud, 2010). A study using aortae from mice expressing a hypertension-inducing PPARγ P457L mutant reports a reduction in neddylated, active Cullin3, along with an elevation of total RhoA protein and ROCK activity. Accordingly, inhibition of Cullin proteins by MLN4924 in wild type mice leads to an increase in arterial pressure *in vivo* and an augmentation of agonist-induced contraction in aortic rings, which is abrogated by blocking ROCK. Thus, loss of Cullin3 may result in disturbed RhoA stability, ROCK activity and elevated contraction and hypertension (Pelham et al., 2012). Interestingly, expression of the adaptor protein RhoBTB1 is abrogated in hypertensive PPARγ P457L mice (Pelham et al., 2012). In a follow-up study, expression of RhoBTB1 in vascular smooth muscle cells (SMC) in these mice restored hypertension, arterial stiffness and impaired agonist-induced vasodilation of basilar arteries and aortae. The authors propose a mechanism, in which RhoBTB1 regulates PDE5 turnover, reducing PDE5 activity, which improves cGMP-dependent SMC relaxation (Mukohda et al., 2019). Hence, Cullin3 limits RhoA-ROCK-mediated contraction and induces SMC relaxation, which may control vasodilation and protect against hypertension.

Mutations in Cullin3 and in the adaptor protein KLHL3 have been discovered in patients affected by pseudohypoaldosteronism type II (PHAII), a syndrome characterized by severe hypertension. Interestingly, all associated Cullin3 mutations cause deletion of exon 9 through a defect in splicing (termed Cul3Δ9) (Boyden et al., 2012). Elevated blood pressure as a consequence of Cul3Δ9 is confirmed in Cullin3^*WT/*Δ9^ mice, along with significant thickening of the vessel wall (Schumacher et al., 2015). Consistently, SMC-specific expression of Cul3Δ9 in transgenic mice leads to increased systolic blood pressure, impaired vasodilation and an augmented contractile response of basilar arteries and the aorta. Inhibition of ROCK restores agonist-induced relaxation (Agbor et al., 2016; Abdel Khalek et al., 2019). Mechanistically, Cul3Δ9 displays impaired binding to Rbx1 in HEK293T cells resulting in attenuated ubiquitination of RhoA (Ibeawuchi et al., 2015; Abdel Khalek et al., 2019). Elevation of RhoA levels is also reported in aortae from mice with SMC-specific expression of Cul3Δ9 (Agbor et al., 2016; Abdel Khalek et al., 2019). Cul3Δ9 forms unstable heterodimers with wild type (WT) Cullin3 leading to disruption of active WT Cullin3 homodimers and a reduction of active neddylated Cullin3 in HEK293T and human aortic SMC (Ibeawuchi et al., 2015; Agbor et al., 2016). These studies suggests that Cul3Δ9 acts dominantly over Cullin3 WT, disrupts its protective effect on blood pressure due to elevated RhoA-induced ROCK activity in SMC and contributes to severe hypertension in PHAII (Ibeawuchi et al., 2015). These findings confirm that Cullin3 regulates blood pressure by controlling RhoA stability.

The above overview underscores the relevance of Cullin3 in different vascular functions, in part through their direct control of Rho GTPases and their regulators. Based on the large number of BTB proteins participating in Cullin3 complexes, it is very likely that we have only detected a small fraction of the complexity and relevance of ubiquitination-based modification of intracellular signaling in the vasculature.

## Author Contributions

FP wrote the manuscript and made the figures. PH edited the manuscript. Both authors contributed to the article and approved the submitted version.

## Conflict of Interest

The authors declare that the research was conducted in the absence of any commercial or financial relationships that could be construed as a potential conflict of interest.

## Abbreviations

BACURD BTB/POZ: domain-containing adaptor for Cul3-mediated RhoA degradation protein
BAZF: B-cell chronic lymphocytic leukemia/lymphoma 6 zinc finger protein
BBS: Bardet-Biedl syndrome
BTB, Bric-à-brac: tramtrack and broad
CBF1: C-promoter binding factor 1
cGMP: cyclic guanosine monophosphate
cIAP/XIAP: cellular and X-linked inhibitor of apoptosis protein
CSN: COP9 signalosome
DAXX: death-domain associated protein
Dll4: Delta-like ligand 4
ECs: endothelial cells
ERRa: estrogen-related receptor α
GABARAP: gamma-aminobutyric acid receptor-associated protein
GAP: GTPase-activating protein
GDI: guanine dissociation inhibitor
GEF: guanine nucleotide exchange factor
HACE1: HECT domain and ankyrin repeat containing E3 ubiquitin protein ligase 1
HEK293T: human embryonic kidney 293 cells expressing SV40 T-antigen
HER2: human epidermal growth factor receptor 2
HUVECs: human umbilical vein endothelial cells
IL-6/8: Interleukin 6/8
K63: lysine 63
KBTBD: Kelch repeat and BTB domain-containing protein
KCTD: potassium (K^+^) channel tetramerization domain
KLHL: Kelch-like protein
MEFs: mouse embryonic fibroblasts
NEDD8: neuronal precursor cell-expressed developmentally down-regulated protein 8
NF κ B: nuclear factor kappa-light-chain-enhancer of activated B cells
NRP1: neuropilin 1
PDE5: phosphodiesterase 5
PDZ-RhoGEF: PDZ-domain containing guanine nucleotide exchange factor for Rho GTPases
PHAII: pseudohypoaldosteronism type II
PPAR γ: peroxisome proliferator-activated receptor gamma
Rbx1: RING box protein
RING: really interesting new gene
ROCK: Rho kinase
SMCs: smooth muscle cells
Smurf1: smad ubiquitination regulatory factor
SPOP: speckle-type POZ (Pox virus and Zinc finger) protein
Tiam1: T-cell Invasion And Migration1
TNFAIP1: TNF alpha induced protein 1
VE-cadherin: vascular endothelial cadherin
VEGF: vascular endothelial growth factor
VEGFR2: vascular endothelial growth factor receptor 2
WT: wild type.
